# Effects of chronic social defeat stress on peripheral leptin and its hypothalamic actions

**DOI:** 10.1186/1471-2202-15-72

**Published:** 2014-06-06

**Authors:** Wataru Iio, Haruyoshi Takagi, Yasuki Ogawa, Takamitsu Tsukahara, Shigeru Chohnan, Atsushi Toyoda

**Affiliations:** 1 Department of Biological Production Science, United Graduate School of Agricultural Science, Tokyo University of Agriculture and Technology, Fuchu, Tokyo 183-8509, Japan; 2 Department of Biological Production Science, College of Agriculture, Ibaraki University, Ami, Ibaraki 300-0393, Japan; 3 Kyoto Institute of Nutrition and Pathology Inc., Ujitawara, Kyoto 610-0231, Japan; 4 Department of Applied Life Science, United Graduate School of Agricultural Science, Tokyo University of Agriculture and Technology, Fuchu, Tokyo 183-8509, Japan; 5 Department of Bioresorce Science, College of Agriculture, Ibaraki University, Ami, Ibaraki 300-0393, Japan

**Keywords:** Anorexia, Chronic social defeat stress, Depression, Hypothalamus, Leptin, Malonyl-CoA

## Abstract

**Background:**

Suppression of body weight and symptom of anorexia are major symptoms of depression. Recently, we reported that chronic social defeat stress (CSDS) induced suppression of body weight gain and anorexic feeding behavior in rats. These abnormalities were the result of disrupted malonyl-coenzyme A (CoA) signaling pathway in the hypothalamus. However, the condition of peripheral leptin and its hypothalamic downstream signal molecules which regulate hypothalamic malonyl-CoA level in the CSDS-exposed rats (CSDS rats) is still unknown.

**Results:**

CSDS rats showed suppressed body weight gain and food intake. The weight of the CSDS rats’ epididymal white adipose tissues was decreased when compared to the control rats. The plasma cholesterol concentration was decreased significantly in the CSDS rats compared to the control rats (*P* < 0.05). The plasma glucose concentration was slightly decreased in the CSDS rats compared to the control rats (*P* < 0.1). The expression of leptin mRNA in epididymal white adipose tissues and the plasma leptin concentration were decreased in CSDS rats. Furthermore, the phosphorylation of the hypothalamic downstream signals of leptin, including extracellular signal-regulated kinase 1/2 (ERK1/2) and signal transducer and activator of transcription 3 (STAT3), was decreased in CSDS rats.

**Conclusions:**

Our results indicated that decreased peripheral leptin expression in CSDS rats could down-regulate the hypothalamic downstream signaling pathways of leptin while suppressed food intake. These data indicate that CSDS induces the down-regulation of hypothalamic AMPK following the elevation of hypothalamic malonyl-CoA levels and is independent of peripheral leptin and glucose.

## Background

Depression causes various health problems, and eating disorders such as loss of appetite are the major symptoms of depression [1,2]. We investigated the molecular mechanisms of depression-induced symptom of anorexia with an animal model of depression. Rats were exposed to CSDS using a resident-intruder paradigm as previously described [3,4]. The CSDS rats showed decreased body weight gain and food intake [3,4]. Because feeding behavior is regulated by the central nervous system, in particular the hypothalamus, we focused on the hypothalamic signal molecules and metabolites that were related to feeding behaviors in CSDS rats [4].

Leptin is known a potent anorexic peptide hormone, and it is synthesized by adipose tissues [5]. In general, a decreased fat volume in the body (or weight) induces the reduction of the peripheral leptin concentration and increases food intake [6]. Conversely, an increased fat volume in the body induces an increased peripheral leptin concentration and reduced food intake [7,8]. The participation of leptin in depression was described in depressive patients and depressive animal models. One study reported that leptin levels did not differ between depressive patients and healthy controls [9]. Other reports described plasma leptin levels that were higher in depressive patients, with a bias in female patients [10,11]. In animal depression models, Lu et al. described subjects with both chronic social defeat stress and chronic mild stress that had decreased plasma leptin concentrations but did not have a change in body weight; they also reported that leptin had an antidepressant-like activity [12,13]. Furthermore, Chuang et al. described that CSDS reduced body weight and plasma leptin concentration [14,15].

Recently, we reported that CSDS rats showed a decrease in food intake and inhibition of body weight gain [4]. Furthermore, CSDS rats showed an increased concentration of hypothalamic malonyl-CoA because of the inhibition of AMPK and the activation of ACC [4]. Hypothalamic malonyl-CoA is also known to play a pivotal role in feeding behavior and malonyl-CoA is synthesized from acetyl-CoA by ACC [16]. ACC is phosphorylated and inactivated by AMPK [16,17]. Malonyl-CoA is a known intermediate in fatty acid biosynthesis and is synthesized from acetyl-CoA by ACC. ACC is phosphorylated and inactivated by AMPK [16,17]. Hypothalamic AMPK is a pivotal protein kinase in the malonyl-CoA signaling pathway, and its activity is inhibited by leptin and glucose [6,16]. In general, rats which showed suppressed body weight gain show reduced peripheral leptin concentration and increased food intake. However, CSDS rats showed suppressed body weight gain and decreased food intake [4]. Therefore, we focused on the peripheral leptin and its hypothalamic downstream signal molecules which are upstream regulators of malonyl-CoA in the CSDS rats.

In this study, we focused on peripheral leptin expression and its downstream hypothalamic signaling pathway and blood plasma components including protein, triglycerides, cholesterol and glucose in CSDS rats to elucidate the effectors for hypothalamic AMPK and malonyl-CoA signaling pathways in CSDS-induced symptom of anorexia.

## Results

### Effects of CSDS on body weight gain and epididymal fat weight

**Figure 1:**
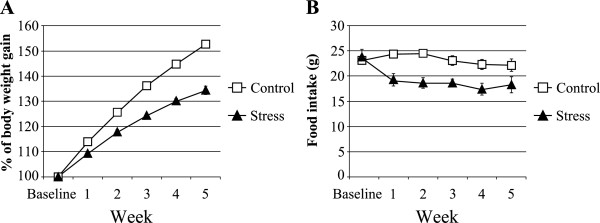
**The effects of chronic social defeat stress on the body weight gain and the food intake. (A)** The body weight gain was calculated relative to the initial (baseline) body weight. Regarding the body weight gain, a two-way repeated measures ANOVA showed that the main effect for stress (*P* < 0.001) and the stress × time interaction (*P* < 0.001) were significant. **(B)** Total food intake during the dark phase. Regarding the food intake, a two-way repeated measures ANOVA showed that the main effect for stress (*P* < 0.05) and the stress × time interaction (*P* < 0.001) were significant. Data represent the mean ± S.E.M. (*n* = 5 / group).

**Figure 2:**
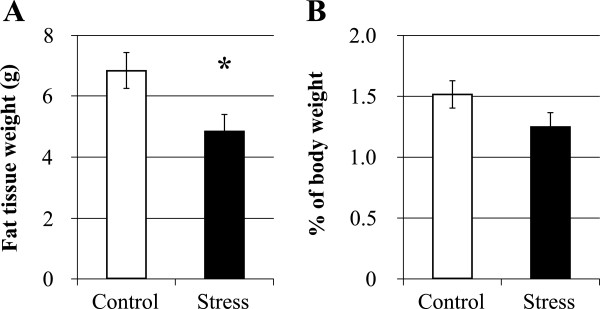
**The effects of chronic social defeat stress on the epididymal fat tissue. (A)** The epididymal fat weight. **(B)** The percentage of fat weight to body weight at the end of the experiment. *P < 0.05 (Student’s *t*-test). Data represent the mean ± S.E.M. (Control, *n* = 8, Stress, *n* = 7).

Body weight was measured at the end of the control phase (baseline) and at weekly intervals during the stress phase. Prior to stress exposure, the body weights of the CSDS and the control rats were similar (Control, 312.2 ± 3.0 g vs. Stress, 306.3 ± 6.5 g, *P* > 0.1). In this study, the CSDS rats showed a suppression in body weight gain compared to the control rats (Figure 1A). A two-way repeated measures ANOVA revealed that stress had a significant effect on body weight gain (*F* (1, 59) = 187.55, *P* < 0.001), and the stress × time interaction was significant (*F* (5, 59) = 68.78, *P* < 0.001). Furthermore, CSDS rats showed decreased food intake during the dark phase (Figure 1B). A two-way repeated measures ANOVA revealed that stress had a significant effect on food intake (*F* (1, 59) = 10.93, *P* < 0.05), and the stress × time interaction was significant (*F* (5, 59) = 5.21, *P* < 0.001). Next, we measured epididymal fat in the CSDS rats. The weight of epididymal fat in the CSDS rats was significantly less than that of the control rats (Control, 6.832 ± 0.588 g vs. Stress, 4.852 ± 0.588 g, *P* < 0.05; Figure 2A). There was no significant difference in the ratio of epididymal fat weight to body weight between the CSDS and the control (Control, 1.515 ± 0.113% vs. Stress, 1.250 ± 0.122%, *P* > 0.1; Figure 2B). These results indicated that the CSDS rats in this study showed suppressed body weight gain and food intake similar to previous our study and decreased epididymal fat in the CSDS rat was occurred with suppressed body weight gain [3,4].

### Effects of CSDS on blood components

We analyzed the total concentration of protein, triglycerides, cholesterol and glucose in the blood plasma. The total protein and glucose concentrations in the plasma of the CSDS rats were only slightly reduced compared to the control rats (Total protein: Control, 5.8 ± 0.1 g/dL vs. Stress, 5.5 ± 0.1 g/dL, *P* < 0.1; Blood glucose: Control, 239.1 ± 24.6 mg/dL vs. Stress, 179.4 ± 13.0 mg/dL, *P* < 0.1; Table 1). The total cholesterol concentration in the plasma of CSDS rats was decreased significantly compared to the control rats (Control, 77.8 ± 1.4 mg/dL vs. Stress, 69.8 ± 1.5 mg/dL, *P* < 0.01; Table 1). However, the plasma triglyceride level was not significantly changed in the CSDS rats (Control, 125.8 ± 31.8 mg/dL vs. Stress, 73.0 ± 13.6 mg/dL, *P* > 0.1; Table 1).

**Table 1:** The effects of chronic social defeat stress on the components of the blood plasma

	**Control**	**Stress**	**P-value**
Blood glucose (mg/dL)	239.1 ± 24.6	179.4 ± 13.0^†^	*P* < 0.1
Total protein (g/dL)	5.8 ± 0.1	5.5 ± 0.1^†^	*P* < 0.1
Triglyceride (mg/dL)	125.8 ± 31.8	73.0 ± 13.6	NS
Total cholesterol (mg/dL)	77.8 ± 1.4	69.8 ± 1.5**	*P* < 0.01

The effects of chronic social defeat stress on the components of the blood plasma. ^†^*P* < 0.1, **P < 0.01 (Student’s *t*-test). NS, not significant. Data represent means ± S.E.M. (*n* = 5 / group).

### Effects of CSDS on leptin signals

**Figure 3:**
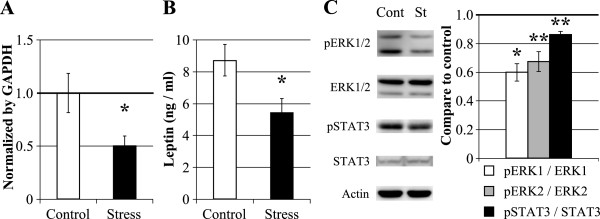
**The effects of chronic social defeat stress on leptin and its hypothalamic downstream signals. (A)** Evaluation of leptin gene expression using real time PCR analysis in the fat tissue (Control, *n* = 8; Stress, *n* = 7). **(B)** Evaluation of leptin peptide expression in the blood plasma (Control, *n* = 8; Stress, *n* = 7). **(C)** Evaluation of the expression and phosphorylation of STAT3 and ERK1/2 in the hypothalamus using western blot analysis. Each band was normalized to actin levels and compared quantitatively using Image J software. (*n* = 4 / group). *P < 0.05, **P < 0.01 (Student’s *t*-test); Cont, Control; St, Stress. Data represent the means ± S.E.M.

Next, we evaluated leptin expression in the white adipose tissue and its hypothalamic intracellular downstream molecules (STAT3 and ERK1/2) under the CSDS condition. We observed that the CSDS rats have significantly lower expression of leptin mRNA in the white adipose tissue than the control rats (Control, 1.000 ± 0.183 vs. Stress, 0.505 ± 0.092, *P* < 0.05; Figure 3A). Secondly, we analyzed the plasma leptin concentration, and we observed that in the CSDS rats, the plasma leptin concentration was significantly decreased (Control, 8.729 ± 0.994 ng/mL vs. Stress, 5.455 ± 0.854 ng/mL, *P* < 0.05; Figure 3B). Moreover, we observed significantly decreased phosphorylation of STAT3 and ERK1/2 in the hypothalamus of CSDS rats. The levels of ERK1/2 and STAT3 protein expression in the hypothalamus were similar between the CSDS and control rats (data not shown), whereas the ratios of phospho-ERK1/2/ERK1/2 and phospho-STAT3/STAT3 were significantly lower in the CSDS rats (phospho-ERK1/ERK1: Control, 1.000 ± 0.135 vs. Stress, 0.594 ± 0.060, *P* < 0.05; phospho-ERK2/ERK2: Control, 1.000 ± 0.033 vs. Stress, 0.674 ± 0.069, *P* < 0.01; phospho-STAT3/STAT3: Control, 1.000 ± 0.011 vs. Stress, 0.859 ± 0.023, *P* < 0.01; Figure 3C). These results indicated that decreased leptin concentration in the plasma was occurred by decreased expression of leptin mRNA in the white adipose tissue. Furthermore, phosphorylation of STAT3 and ERK1/2 in the hypothalamus was not conflict with leptin concentration in the plasma.

## Discussion

Depression often induces symptom of anorexia and a severe reduction in the body weight of subjects with some disrupted hypothalamic regulation feeding behaviors [1,2]. To elucidate the mechanism of loss of appetite induced by CSDS and depression, we evaluated the levels of peripheral leptin, its downstream hypothalamic signals and blood components such as protein, triglycerides, cholesterol and glucose in CSDS rats that were obtained using a resident-intruder paradigm.

In this study, CSDS significantly reduced body weight gain and epididymal fat, but it did not specifically induce the accumulation of epididymal fat. There are some reports describing the relationship between CSDS and body weight change. Body weight loss was observed in CSDS mice obtained by a ten-day session of a social defeat paradigm with resident mice (CD-1) and intruder mice (C57BL6/J) [15]. The intruder’s body weight was quickly reduced with exposure to the social defeat paradigm, but after a ten-day session of the CSDS program, the body weight of the defeated intruders increased faster than the controls [15]. Another report described that the duration of the physical contacts in the CSDS paradigm is critical for stress-induced body weight change. Savignac et al. developed a short-term version of the CSDS paradigm that allow only one contact between the resident and intruder [18]. In this short-term version of the CSDS paradigm, the C57BL/6 J intruder’s body weight increased [18]. In our study, the CSDS rats did not have body weight loss, but they instead displayed reduced body weight gain (Figure 1A) [4]. This discrepancy may be due to differences of experimental animals and CSDS paradigms. In the future, the patterns of body weight changes in depressive patients should be compared with animal models of depression to understand the roles of weight loss and obesity in depression.

Depression also affects the concentration of some blood components [14]. Using metabolic analyses, we found that CSDS induces various changes of the metabolites in the blood plasma (data not shown). In this study, we analyzed plasma glucose, triglycerides, cholesterol, and protein levels using conventional biochemical techniques. Some studies reported that low concentrations of cholesterol are associated with mood disorders such as major depression [19-23]. Engelberg reported that low concentrations of serum cholesterol may decrease serotonin concentrations in the brain and fail to suppress aggressive behavior [24]. Furthermore, leptin and cholesterol are known to be decreased in the depressive patients [21,23]. Moreover, leptin is also related to decreased serotonin concentration in the depressive patients through regulating serotonin turnover [21,25]. Thus, our CSDS rats are a suitable model of animal depression for understanding cholesterol metabolism during depression. Indeed, Chuang et al. reported that CSDS induced disrupted regulation of lipid synthesis. Their model which is increased body weight by exposed CSDS increased expression of genes involved in the fatty acid synthesis and decreased expression of genes involved in the fatty acid utilization in the liver. However, they reported that CSDS and high-fat diet induced increased total cholesterol and no changed triglycerides in the tissue [14]. Therefore, we need to investigate expression of genes related lipid synthesis.

Next, we focused on peripheral leptin expression and its hypothalamic downstream signals in CSDS rats. Leptin is a potent anorexigenic peptide hormone and is synthesized by white adipose tissues. Peripheral leptin can enter into the brain tissues through the brain blood barrier and activate the STAT3 and MAPK cascades by binding leptin receptors [26,27]. Leptin is one of the upstream ligands for the malonyl-CoA signaling pathway in the hypothalamus. The concentration of hypothalamic malonyl-CoA regulates the expression of neuropeptides and controls the feeding behavior [16]. Administration of C75, a fatty acid synthase inhibitor, induced an increase in the malonyl-CoA concentration, affected the expression of neuropeptides in the hypothalamus and decreased food intake [26,28]. Malonyl-CoA is synthesized from acetyl-CoA by ACC, and the ACC activity is regulated by AMPK. Therefore, leptin and malonyl-CoA signaling pathways in the hypothalamus play a pivotal role in feeding behaviors. Moreover, the relationship between leptin and mood disorders was investigated. Depressive patients and socially defeated rats had decreased leptin concentrations in their blood plasma [13,29]. Recently, we reported that CSDS rats showed suppressed food intake and body weight gain following an increase in their hypothalamic malonyl-CoA concentration [4]. Therefore, we focused on the mechanism of the elevation of hypothalamic malonyl-CoA levels in CSDS-induced symptom of anorexia. We first observed peripheral leptin expression and its hypothalamic action. The expression of leptin mRNA in the CSDS rat fat tissue was significantly lower than that of the control rats (Figure 3A). Furthermore, the leptin blood plasma concentration in the CSDS rats was significantly lower (Figure 3B). Previous studies indicated that rats exposed to ten-day sessions of social defeat stress showed decreased plasma leptin concentrations; however, the stressed rats did not show any body weight change [19,29]. In depressive patients, decreased plasma leptin concentrations and no significant body mass indices changes were observed [29]. CSDS most likely suppressed the expression of the leptin gene in the fat tissue and decreased the plasma leptin concentration. The mechanisms of its inhibition by CSDS are unknown. Leptin is also known to regulate the renin-angiotensin-aldosterone system which showed abnormality in depressive patient and anorexic patient [30-32]. Furthermore, leptin inhibits adrenocortical steroid production including aldosterone and corticosterone [31]. Therefore, there is a possibility that our CSDS rats also indicate abnormality of the renin-angiotensin-aldosterone system. Furthermore, we evaluated the downstream hypothalamic signal transduction of leptin in CSDS rats. Hypothalamic phosphorylation of STAT3 and ERK1/2 in CSDS rats were not conflict with peripheral leptin levels in the CSDS rats. As described by Gao et al., intraventricular administration of leptin increased the phosphorylation of hypothalamic STAT3 and decreased that of AMPK and ACC, following an increase in the malonyl-CoA concentration and a decrease in food intake [6]. However, CSDS rats showed decreased food intake during the dark phase (Figure 1B). Therefore, CSDS may disrupt some hypothalamic signaling pathways from leptin receptors to AMPK. Our previous data showed decreased hypothalamic phospho-AMPK and food intake in CSDS rats [4]. Adiponectin is also known to relate to feeding behavior and loss of appetite [33,34]. Therefore, the activity of adiponectin in CSDS rats should be investigated in the future studies.

## Conclusions

Symptom of anorexia was induced in our CSDS rats, and they had suppressed body weight gain following a decrease of phospho-AMPK in the hypothalamus. We also observed the down-regulation of peripheral leptin and hypothalamic downstream signals of the leptin receptor. Furthermore, the blood glucose concentration had a tendency to decrease in CSDS rats. Thus, the inhibition of AMPK and the elevation of the malonyl-CoA concentration in the hypothalamus of CSDS rats as previously reported were independent of peripheral glucose and leptin signals. Together, these data suggest that CSDS downregulates hypothalamic AMPK following elevation of malonyl-CoA via some unknown factors that should be elucidated in future studies.

## Methods

### Ethics statement

This study was reviewed and approved by the guidelines of the Animal Care and Use Committee of Ibaraki University (No.84 and No.114) and conformed to the Ministry of Education, Culture, Sports, Science and Technology, Japan (Notification, No.71).

### Animals

The detailed experimental animals and designs have been described previously [4]. To obtain CSDS rats, the resident-intruder paradigm was performed as described. Eight-week-old male Wistar rats were purchased from Charles River (Kanagawa, Japan) and were housed individually at room temperature (22 ± 1°C) with exposure to light from 6:00 to 18:00 and *ad libitum* access to food and water. Food intake was measured using a food intake monitor (O’Hara & Co., Ltd.). After their arrival, the rats were handled daily for 1 week to habituate them to the environment; they were then used as intruders. Twelve-week-old male Wistar rats from our colonies were used as residents and housed large cage (L60 cm × W45 cm × H45 cm) with same age sterilized female rat. The female rats were removed from their home cage, and an intruder rat was introduced into each resident’s home cage for up to 1 hour. Within the first 10 minutes, the intruder was usually attacked and defeated by the resident and would show subordinate behaviors, including vocalization, jumping, freezing and a submissive posture [35]. After the intruder displayed a submissive posture, the intruder was immediately removed and kept in a wire-mesh cage within the resident’s home cage for the remainder of the hour. Rats from the stress group were subjected to this social defeat procedure on a daily basis for 5 weeks. Body weight was measured at the end of the control phase (baseline) and at weekly intervals during the stress phase. At the end of the program, the rats which fed *ad libitum* were anesthetized and decapitated in the light phase, between 10:00 to 14:00, for the biochemical analysis described below [4].

### cDNA preparation and real time PCR

Adipose tissues were collected from epididymides and weighed. The total RNA was extracted using the LiCl/Urea method. cDNA was synthesized using ReverTraAce (Toyobo, Osaka, Japan). Real-time PCR was performed using a Thermal Cycler Dice Real Time System Single (Takara, Osaka, Japan). The GAPDH gene was amplified from all the samples to normalize expression. The following primers were used: Rat gapdh, (AB017801.1, 73–92), 5′-GTATTGGGCGCCTGGTCACC-3′ and (253–274) 5′-CGCTCCTGGAAGATGGTGATGG-3′; Rat leptin, (NM013076.3, 362–381) 5′-GAGACCTCCTCCATGTGCTG-3′ and (529–548) 5′-CATTCAGGGCTAAGGTCCAA-3′.

### Measurement of the leptin concentration in the plasma

The rat blood from the abdominal aorta was collected in heparinized test tubes. These test tubes were centrifuged at 1,000 × *g* for 15 minutes at 4°C, and the plasma was collected. The leptin concentration in the plasma was measured using a Rat Leptin ELISA Kit (Morinaga, Kanagawa, Japan). The detection was performed using a Wallac 1420 ARVOsx (Wallac, Tokyo, Japan).

### Blood analysis

The blood plasma glucose concentration was measured using a Glucose CII-Test kit (Wako Pure Chemicals, Osaka, Japan). The concentrations of triglyceride, total cholesterol and total protein in the plasma were determined by the Japan Clinical Laboratories (Kyoto, Japan).

### Protein preparation and western blotting

The rat brains were rapidly removed and chilled on ice, and the hypothalami were retrieved. The tissue was homogenized in ice-cold RIPA buffer with a Polytron homogenizer (IKA Japan, Osaka, Japan). The homogenate was centrifuged at 800 × *g* for 15 minutes at 4°C, and the supernatant was collected. Proteins were detected using ECL prime western blotting detection reagents (GE Healthcare, Tokyo, Japan) and a LAS-3000 mini (FUJIFILM, Kanagawa, Japan).

The following primary antibodies were used as described below: anti-actin (Santa Cruz), anti-STAT3 (Usbiological), anti-phospho-STAT 3 (Cell Signaling), anti-ERK1/2 (Invitrogen) and anti-phospho-ERK1/2 (Cell Signaling). The western blotting results were analyzed quantitatively using Image J.

### Statistical analysis

Data were analyzed using Excel Toukei 2006 for Windows (Social Survey Research Information Co., Ltd. Tokyo, Japan). Body weight gain data was analyzed using two-way repeated measures ANOVAs. Other data was analyzed by Student’s *t*-test.

## Abbreviations

CSDS: Chronic social defeat stress; CSDS rats: CSDS-exposed rats; CoA: Coenzyme A; ACC: Acetyl-CoA carboxylase; AMPK: Adenosine monophosphate-activated protein kinase; STAT3: Signal transducer and activator of transcription 3; ERK1/2: Extracellular signal-regulated kinase 1/2.

## Competing interests

The authors declare that they have no competing interest.

## Authors’ contributions

WI and AT planed this research and wrote this manuscript. WI performed the animal experiments, behavioral tests and biochemical analysis. HT, YO, TT and SC performed molecular and biochemical analyzes. All authors read and approved the final manuscript.
